# Down‐regulation of aquaporin 9 gene transcription by 10‐hydroxy‐2‐decenoic acid: A major fatty acid in royal jelly

**DOI:** 10.1002/fsn3.1246

**Published:** 2019-10-21

**Authors:** Shigeyuki Usui, Midori Soda, Kazuhiro Iguchi, Naohito Abe, Masayoshi Oyama, Tatsushi Nakayama, Kiyoyuki Kitaichi

**Affiliations:** ^1^ Department of Biomedical Pharmaceutics Gifu Pharmaceutical University Gifu Japan; ^2^ Instrumental Analysis Center Gifu Pharmaceutical University Gifu Japan; ^3^ Department of Community Pharmacy Gifu Pharmaceutical University Gifu Japan; ^4^ Department of Pharmacognosy Gifu Pharmaceutical University Gifu Japan

**Keywords:** 10‐hydroxy‐2‐decenoic acid, Akt, AMP‐activated protein kinase (AMPK), aquaporin 9 (AQP9), forkhead box a2 (Foxa2), insulin

## Abstract

10‐Hydroxy‐*trans*‐2‐decenoic acid (10H2DA) is a unique lipid component of royal jelly produced by worker honeybees that exerts insulin‐like effects. We herein investigated the effects of 10H2DA on the gene expression of aquaporin 9 (AQP9), which functions as a glycerol transporter in the liver, to clarify whether 10H2DA modulates energy metabolism. 10H2DA suppressed AQP9 gene expression in HepG2 cells by promoting the phosphorylation of Akt and AMP‐activated protein kinase (AMPK). This suppression was partially recovered by the treatment of cells with inhibitors for Akt and AMPK. Based on the result showing that leptomycin B partially recovered the suppression of AQP9 gene expression, 10H2DA inhibited the expression of Foxa2, a transcription factor for the AQP9 gene, and also induced its nuclear exclusion. Although 10H2DA up‐regulated phosphoenolpyruvate carboxykinase and glucose‐6‐phosphatase gene expression, this was suppressed through the modulation of Foxa2 by insulin. These results suggest that 10H2DA suppresses AQP9 gene expression through the phosphorylation of Akt and AMPK and down‐regulation of Foxa2 expression.

## 1 INTRODUCTION

Aquaporin 9 (AQP9), which belongs to the aquaglyceroporin family including AQP3, 7, and 10, is mainly expressed in the human liver, brain, testis, and white blood cells, and functions as a unique glycerol channel in the liver (Calamita et al., 2012; Tsukaguchi et al., 1998; Tsukaguchi, Weremowicz, Morton, & Hediger, 1999). Since glycerol is utilized as a source material for glyco‐ and lipid metabolism, aquaglyceroporins may participate in these metabolic pathways by transporting glycerol. The knockout of AQP9 was previously reported to significantly reduce glucose levels and increase glycerol levels in the blood of obese model mice carrying a mutated leptin receptor (Rojek et al., 2007). Insulin reduces blood glucose levels by modulating particular enzymes, transporters, and transcriptional regulators involved in glucose metabolism. AQP3, 7, and 9 gene expression was previously reported to be suppressed by insulin through the activation of Akt (Kishida et al., 2001; Kuriyama et al., 2002). Forkhead box a2 (Foxa2/HNF3β), a transcriptional regulator, has been identified as a contributing factor to the suppression of AQP3 and 9 expression by insulin (Higuchi et al., 2007; Yokoyama, Iguchi, Usui, & Hirano, 2011). AMP‐activated protein kinase (AMPK) has been suggested to function as an energy sensor in the maintenance of the homeostasis of glyco‐ and lipid metabolism (Kahn, Alquier, Carling, & Hardie, 2005). The activation of AMPK by 5‐aminoimidazole‐4‐carboxamide‐1‐β‐D‐ribonucleoside (AICAR) was previously shown to suppress AQP9 expression via the phosphorylation of Akt followed by the phosphorylation and nuclear exclusion of Foxa2 (Yokoyama et al., 2011).

10‐Hydroxy‐*trans*‐2‐decenoic acid (10H2DA), 3,10‐dihydroxydecanoic acid, and sebacic acid are unique medium chain fatty acids in royal jelly (Lercker, Capella, Conte, Ruini, & Giordani, 1981). 10H2DA has been reported to exhibit various pharmacological activities such as antitumor (Townsend et al., 1960), antibiotic (Blum, Novak, & Taber, 1959), neuron proliferator (Hattori, Nomoto, Fukumitsu, Mishima, & Furukawa, 2007), immunomodulator (Takahashi, Sugiyama, Tokoro, Neri, & Mori, 2012), and anti‐rheumatic effects (Yang et al., 2010). Some of the mechanisms by which 10H2DA exerts these effects were recently elucidated. For example, 10H2DA inhibited the LPS‐induced activation via specific reductions in LPS‐induced IκB‐ζ expression and also suppressed LPS‐induced IL‐6 production in murine macrophage‐derived RAW264 cells (Sugiyama et al., 2012). 10H2DA suppressed the mRNA expression of matrix metalloproteinases by blocking p38 kinase and c‐Jun N‐terminal kinase–activator protein‐1 signaling pathways in rheumatoid arthritis synovial fibroblasts (Wang et al., 2012). It has also been shown to modulate the recruitment of estrogen receptors α and β, and β only to the pS2 promoter in the presence and absence of estradiol, respectively, in MCF‐7 cells (Moutsatsou et al., 2010).

Previous studies reported that royal jelly exhibited insulin‐like activity as a hypoglycemic effect (Münstedt, Bargello, & Hauenschild, 2009; Nomura et al., 2007; Zamami et al., 2008). Kameda, Chikaki, Morimoto, Jiang, and Okuda (1996) demonstrated that 10H2DA inhibited lipolysis, stimulated lipogenesis, and exerted insulin‐like effects. Thus, 10H2DA is a candidate component in royal jelly for its hypoglycemic effects. We initially investigated whether 10H2DA affected AQP9 mRNA expression because AQP9 is permeable to glycerol in the liver and glycerol is a source material for glyco‐ and lipid metabolism. The results obtained showed that 10H2DA suppressed AQP9 mRNA expression. We herein elucidated the mechanisms by which 10H2DA inhibits AQP9 mRNA expression.

## 2 MATERIALS AND METHODS

### 2.1 Materials

10H2DA was purchased from Alfresa Pharma, and royal jelly was provided by Api Co., Ltd.. Compound C and Akt 1/2 inhibitor were purchased from Calbiochem‐Merck and Wako Pure Chemicals, respectively. TRIzol, an Oligo(dT)_12‐18_ primer, and SuperScript III reverse transcriptase were purchased from Invitrogen Corp.. DNA‐manipulating enzymes were obtained from Promega Corp.. SYBR Premix Ex Taq, Amersham ECL Plus Western blotting detection reagents, and Immobilon transfer membranes were purchased from Takara Bio, Inc., GE Healthcare UK Ltd., and Millipore Corp., respectively. Anti‐phospho‐PKB (Thr‐308) and anti‐human poly ADP‐ribose polymerase (PARP) antibodies were purchased from Sigma‐Aldrich, Inc., and BD Biosciences, respectively. Anti‐phospho‐Akt (Ser‐473), anti‐Akt, anti‐phospho‐AMPKα (Thr‐172), and AMPKα antibodies were obtained from Cell Signaling Technology, Inc.. An anti‐mouse light chain and anti‐Foxa2 antibodies were obtained from Millipore Corp. and Abcam Co., Ltd., respectively. All other chemicals and reagents were of analytical grade.

### 2.2 Cell culture and treatment with 10H2DA

HepG2 cells were maintained in Dulbecco's Modified Eagle's Medium (DMEM) supplemented with 10% fetal bovine serum (FBS), 100 µg/ml streptomycin, and 100 units/ml penicillin. Cells were plated on a 24‐well culture plate at a density of 2 × 10^5^ cells/well and incubated in a CO_2_ incubator for 24 hr. Cells were washed once with phosphate‐buffered saline (PBS) and then treated with 10H2DA. 10H2DA was neutralized with 0.1 M NaOH before being added to the culture medium.

### 2.3 Real‐time reverse transcription–polymerase chain reaction (RT‐PCR)

Total RNA was extracted from HepG2 cells with TRIzol reagent. First‐strand cDNA was prepared from 5 µg total RNA with the Oligo(dT)_12‐18_ primer and SuperScript III reverse transcriptase and was used as a template. Real‐time RT‐PCR was performed with the specific primers listed in Table 1 and SYBR Premix using the Thermal Cycler Dice Real Time System (TAKARA Bio, Inc.). The cycle threshold signal was used to measure RT‐PCR signals. At the end of PCR, a dissociation curve was produced to examine the specificity of the PCR product. The β2‐microglobulin housekeeping gene was used to normalize target mRNA expression.

**Table 1:** Sequences of oligonucleotide primers and conditions for real‐time RT‐PCR

Gene (accession number)	Primers		PCR condition		Cycles
Aquaporin 9 (NM_020980)	Forward	5′‐TGGCAACATACCCAGCTCCG‐3′	94°C	30 s	35
	Reverse	5′‐CAATGGGCTCTAGGCCTCTG‐3′	57°C	30 s	
			72°C	30 s	
Foxa2 (NM_021784)	Forward	5′‐ACACCACTACGCCTTCAACC‐3′	94°C	30 s	27
	Reverse	5′‐GGGGTAGTGCATCACCTGTT‐3′	60°C	30 s	
			72°C	30 s	
β2‐microglobulin (NM_004048)	Forward	5′‐GGTTTCATCCATCCGACATT‐3′	94°C	30 s	22
	Reverse	5′‐CGGCAGGCATACTCATCTTT‐3′	60°C	30 s	
			72°C	30 s	

### 2.4 Western blot analysis

HepG2 cells treated with or without 10H2DA were lysed with lysis buffer consisting of 20 mM Tris/HCl, pH 7.5, 250 mM sodium chloride, 1 mM EDTA, 1 mM EGTA, 1% (w/v) Triton X‐100, 10% (w/v) glycerol, 1 mM dithiothreitol, 0.2 mM PMSF, 0.5 µg/ml leupeptin, 1 µg/ml pepstatin, 2 mM sodium orthovanadate, 2 mM β‐glycerophosphate, and 5 mM sodium fluoride. Protein concentrations in lysates were measured using the Bradford assay with BSA as a standard and Bio‐Rad protein assay reagent. Twenty micrograms of protein were boiled for 5 min with 1% sodium dodecyl sulfate (SDS) and 4 mM 2‐mercaptoethanol and then loaded onto a 12% SDS‐polyacrylamide gel. SDS‐polyacrylamide gel electrophoresis (SDS‐PAGE) was then conducted according to the method of Laemmli (1970). Western blotting was performed by the method of Towbin, Staehelin, and Gordon (1979). After proteins in the gel had been electroblotted onto an Immobilon membrane, the desired protein was probed with the specific primary antibody followed by the peroxidase‐conjugated secondary antibody. Peroxidase activity was detected with an Amersham ECL Plus Western blotting detection system according to the manufacturer's instructions using LAS‐3000 UV mini (Fujifilm Corp.).

### 2.5 Statistical analysis

The significance of differences between two groups and multiple groups was assessed using Student's *t* test and a one‐way analysis of variance followed by Dunnett's test, respectively.

## 3 RESULTS

### 3.1 Effects of 10H2DA on AQP9 mRNA expression

**Figure 1:**
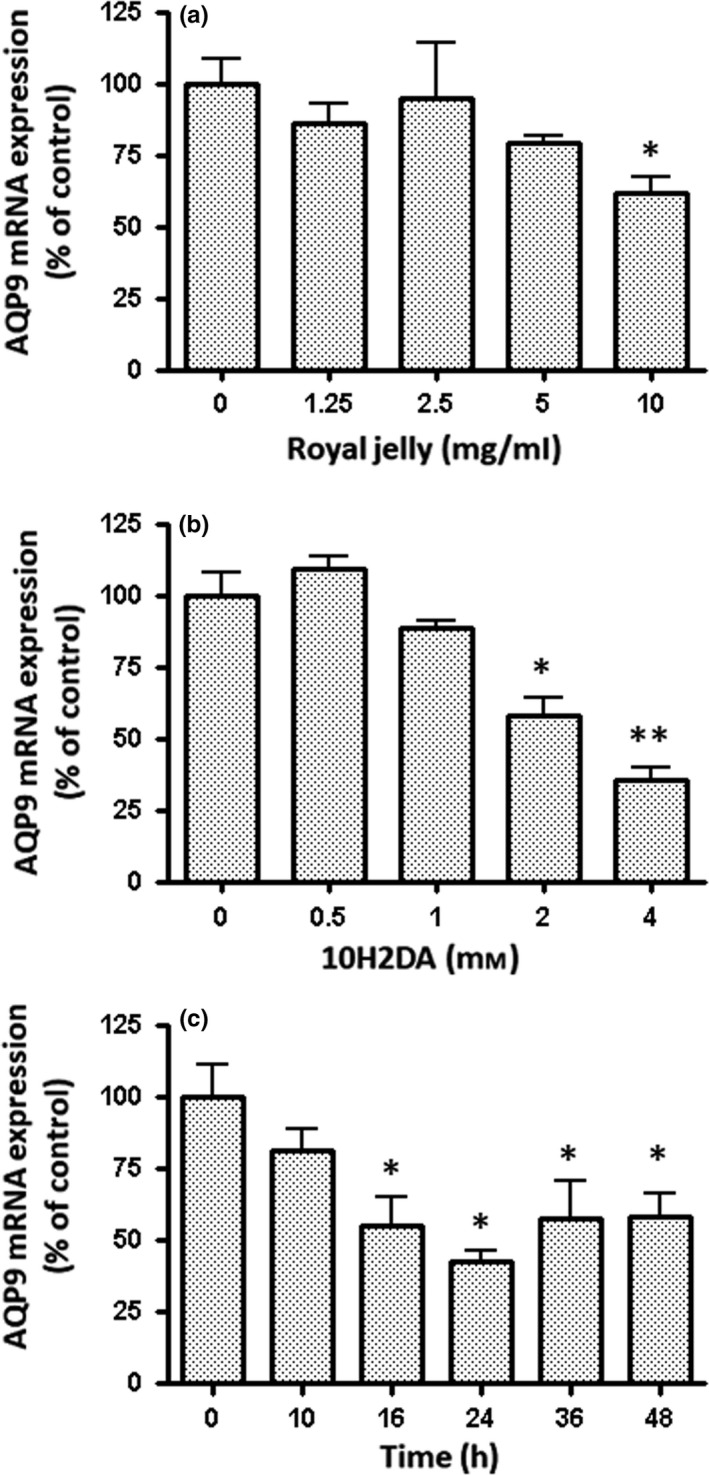
Suppression of AQP9 gene expression by royal jelly and 10‐hydroxy‐2‐decenoic acid (10H2DA). HepG2 cells were incubated with various concentrations of (a) royal jelly and (b) 10H2DA for 24 hr and (c) 4 mM 10H2DA for the period indicated. Cells incubated without royal jelly and 10H2DA were used as a control. AQP9 mRNA expression was quantified by real‐time RT‐PCR after total RNA was extracted, and first‐strand cDNAs were synthesized. Results were normalized with β2‐microglobulin mRNA levels, and the mRNA level of the control was taken as 100%. Data show the mean ± *SD* of five experiments. ^*^ *p* < .05, ^**^ *p* < .01 versus control

We examined whether 10H2DA and royal jelly affected AQP9 mRNA expression in HepG2 cells. AQP9 mRNA expression was significantly down‐regulated in HepG2 cells treated with 10 mg/ml royal jelly and 2 and 4 mM 10H2DA for 24 hr (Figure 1a and b). When HepG2 cells were incubated with 4 mM 10H2DA for the period indicated, AQP9 mRNA expression levels were significantly decreased for 16–48 hr (Figure 1c). High concentrations of 10H2DA were needed to induce this effect in HepG2 cells.

### 3.2 Phosphorylation of AMPK and Akt by 10H2DA

**Figure 2:**
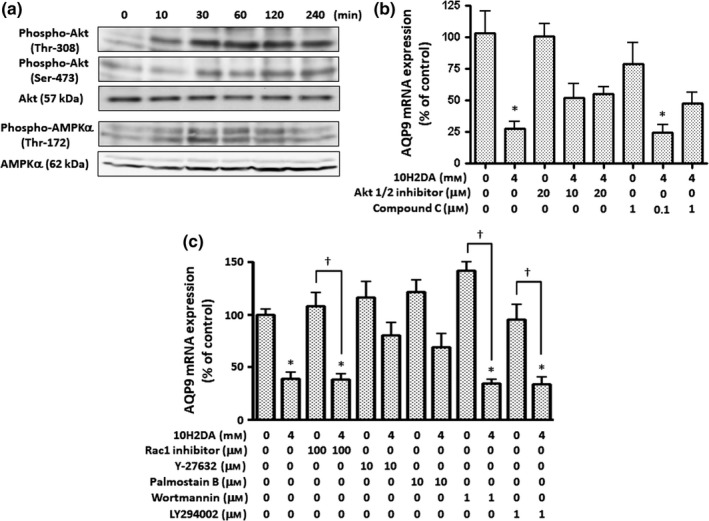
Activation of kinases by 10H2DA. (a) HepG2 cells were incubated with 4 mM 10H2DA for the period indicated. Cell lysates were prepared with lysis buffer containing SDS and 2‐mercaptoethanol, and 20 µg protein from the lysate was separated by electrophoresis with a 12% SDS‐polyacrylamide gel. After proteins in the gel were electroblotted on a PVDF membrane, phosphorylated Akt (Thr‐308), phosphorylated Akt (Ser‐473), Akt, phosphorylated AMPKα (Thr‐172), and AMPKα were probed with specific antibodies and visualized using a secondary antibody‐peroxidase conjugate and the ECL system. (b) and (c) HepG2 cells were pre‐incubated with various inhibitors for 1 hr and then treated with or without 10H2DA for 24 hr. Cells incubated without 10H2DA, and inhibitors were used as a control. AQP9 mRNA expression was quantified by real‐time RT‐PCR. Results were normalized with β2‐microglobulin mRNA levels and the mRNA level of the control was taken as 100%. Data show the mean ± *SD* of five experiments. ^*^ *p* < .05 versus control. ^†^ *p* < .05 versus control with an inhibitor added

We previously reported that 5‐aminoimidazole‐4‐carboxamide‐1‐β‐D‐ribofuranoside (AICAR), an AMP‐activated protein kinase (AMPK) activator, induced the phosphorylation of AMPK and Akt, and down‐regulated AQP9 gene expression in HepG2 cells (Yokoyama et al., 2011). Since AQP9 gene expression was down‐regulated by the treatment with 10H2DA, we investigated whether 10H2DA induced the phosphorylation of these kinases in HepG2 cells (Figure 2a). In HepG2 cells treated with 4 mM 10H2DA, the phosphorylation of AMPKα ‐Thr172 was observed for 10–120 min, with maximum phosphorylation being noted at approximately 30 min, while that of Akt‐Thr308 and Akt‐Ser473 was noted for 10–240 min with maximum phosphorylation occurring at approximately 60 min. Kinase inhibitors were used to examine whether the phosphorylation of these kinases suppressed 10H2DA‐induced AQP9 mRNA expression (Figure 2b). Compound C and Akt 1/2 inhibitor, inhibitors of AMPK and Akt, respectively, achieved the partial remission, but not full recovery of the suppression of AQP9 mRNA expression by 4 mM 10H2DA. This result suggested that the mechanisms by which 10H2DA suppresses AQP9 mRNA expression are based not only on the phosphorylation of these kinases, but also on the other effects of 10H2DA. Since 10H2DA promoted the phosphorylation of Akt, we attempted to elucidate the mechanisms by which 10H2DA induced this phosphorylation, in more detail. Rac1 inhibitor, Y27632, palmostatin B, wortmannin, and LY294002 for Rac1, and Rho‐associated coiled‐coil forming kinase (ROCK), β‐lactone acyl protein thioesterase 1 (APT1), and phosphoinositide 3‐kinase (PI3K) were used to examine the signaling pathways related to the rat sarcoma (Ras) protein, low‐molecular‐weight GTPase, and G protein‐coupled receptor (Figure 2c). Y‐27632 and palmostatin B partially recovered the suppression of AQP9 mRNA expression by 10H2DA, whereas the Rac1 and PI3K inhibitors had no effect. These results suggest that Rho kinase and N‐Ras participate in the signaling pathway triggered by 10H2DA in HepG2 cells and also that Akt is not phosphorylated by the activation of PI3K in the pathway.

### 3.3 Effects of 10H2DA on Foxa2 expression

**Figure 3:**
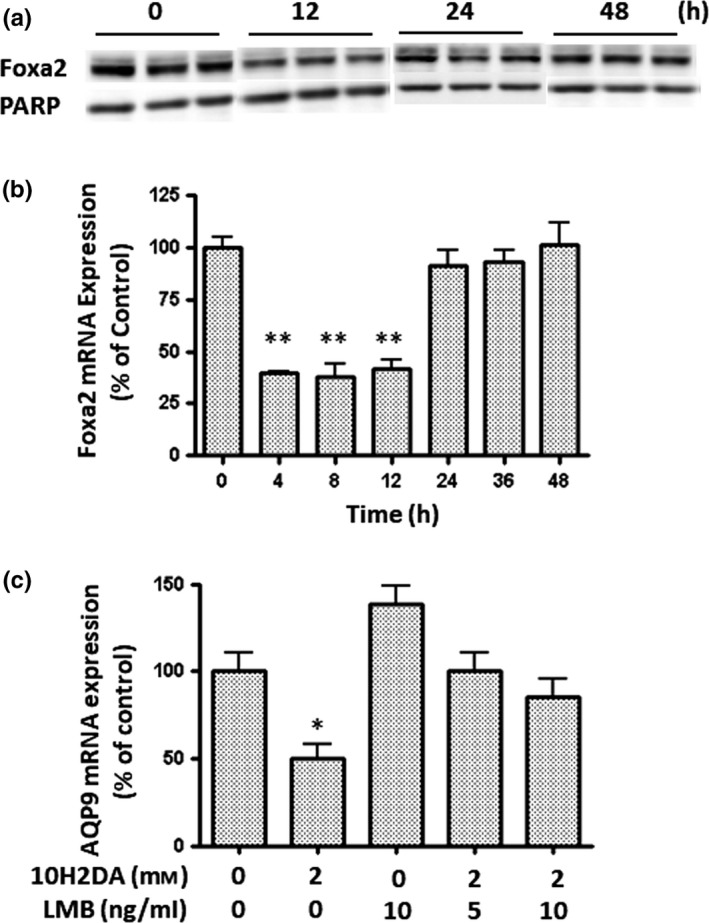
Effects of 10H2DA on transcriptional regulation via forkhead box a2 (Foxa2). (a) SDS‐PAGE and Western blotting were performed with 20 µg of the cell lysate from HepG2 cells treated with 4 mM 10H2DA for the period indicated under the same conditions as those described in Figure 2. After proteins in the gel were electroblotted on a PVDF membrane, Foxa2 and poly ADP‐ribose polymerase (PARP) were probed with specific antibodies and visualized using a secondary antibody‐peroxidase conjugate and the ECL system. (b) HepG2 cells were incubated with 4 mM 10H2DA for the period indicated, (c) were pre‐incubated with the indicated concentration of leptomycin B (LMB) for 1 hr, and were then treated with or without 10H2DA for 24 hr. Cells incubated without 10H2DA and LMB were used as a control. Foxa2 and AQP9 mRNA expression was quantified by real‐time RT‐PCR. Results were normalized with β2‐microglobulin mRNA levels and the mRNA level of the control was taken as 100%. Data show the mean ± *SD* of five experiments. ^*^ *p* < .05, ^**^ *p* < .01 versus control

AMPK activation (Yokoyama et al., 2011) and insulin signaling (Howell & Stoffel, 2009) have been reported to induce the phosphorylation and nuclear exclusion of Foxa2 via the phosphorylation of Akt. Although the phosphorylation of AMPK and Akt was observed following incubations with 10H2DA, as shown in Figure 2, AQP9 mRNA expression remained partially suppressed by treatments with the inhibitors of these kinases. Therefore, we speculated that a mechanism other than the phosphorylation and nuclear exclusion of Foxa2 suppresses AQP9 mRNA expression. Foxa2 protein and mRNA expression was suppressed by the treatment with 4 mM 10H2DA, with a lag time of approximately 12 hr (Figure 3a and b). The suppression of Foxa2 mRNA expression by 10H2DA was observed earlier than that of AQP9 mRNA expression. Foxa2 contains a functional nuclear export signal (NES) and is excluded from the nucleus via a chromosome region maintenance (CRM) 1‐dependent pathway in response to insulin signaling (Howell & Stoffel, 2009). Therefore, the CRM1 inhibitor, leptomycin B (LMB), was employed in the present study to suppress AQP9 mRNA expression via the regulation of Foxa2 triggered by 10H2DA (Figure 3c). Since Foxa2 was retained in nuclei by the inhibition of CRM1 with leptomycin B, AQP9 mRNA expression levels were slightly higher following the treatment with this inhibitor than control levels. When HepG2 cells were treated with 2 mM 10H2DA in the presence of this inhibitor, AQP9 mRNA expression levels were similar to those of the control. However, these expression levels were lower than those without 10H2DA. These results suggest that 10H2DA induces the nuclear exclusion of Foxa2 and suppression of Foxa2 expression.

### 3.4 Differences from insulin

**Figure 4:**
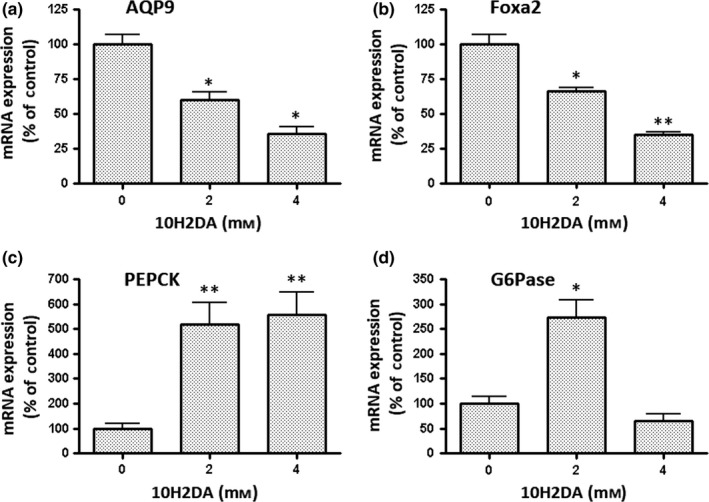
Effects of 10H2DA on the expression of various genes. HepG2 cells were incubated with the indicated concentration of 10H2DA for 24 hr. Cells incubated without 10H2DA, and inhibitors were used as a control. The mRNA expression of (a) AQP9, (b) Foxa2, (c) phosphoenolpyruvate carboxykinase (PEPCK), and (d) glucose‐6‐phosphatase (G6Pase) was quantified by real‐time RT‐PCR. Results were normalized with β2‐microglobulin mRNA levels, and the mRNA level of the control was taken as 100%. Data show the mean ± *SD* of five experiments. ^*^ *p* < .05, ^**^ *p* < .01 versus control

Wolfrum, Besser, Luca, and Stoffel (2003) demonstrated that insulin suppressed phosphoenolpyruvate carboxykinase (PEPCK) and glucose‐6‐phosphatase (G6Pase) gene expression in glycometabolism. This suppression was based on the phosphorylation and nuclear exclusion of Foxa2 in response to PI3K‐Akt signaling by insulin. Since 10H2DA suppressed AQP9 mRNA expression via Foxa2, we investigated whether 10H2DA affected the gene expression of these enzymes and compared its effects to those of regulation by insulin (Figure 4). The gene expression of these enzymes, except for G6Pase, was up‐regulated by 4 mM 10H2DA. These results suggest that the effects of 10H2DA on glycometabolism differ from those of insulin, even though AQP9 gene expression was down‐regulated by insulin and 10H2DA.

## 4 DISCUSSION

In the present study, we demonstrated that 10H2DA suppressed AQP9 gene expression in HepG2 cells by phosphorylating AMPK and Akt and also inhibited Foxa2 gene expression. Foxa2 has been identified as a transcriptional factor for the gene expression of AQP3 (Higuchi et al., 2007) and 9 (Yokoyama et al., 2011). The phosphorylation of Akt by insulin and AICAR, an AMPK activator, induced the phosphorylation and nuclear exclusion of Foxa2, which ultimately suppressed target gene expression (Wolfrum et al., 2003; Yokoyama et al., 2011). 10H2DA may exert similar effects on Foxa2, resulting in the transcriptional repression of the AQP9 gene. Furthermore, 10H2DA down‐regulated Foxa2 gene expression. Since AQP9 contributes to glyco‐ and lipid metabolism by transporting glycerol in the liver, the suppression of AQP9 gene expression by 10H2DA may have a negative impact on this supply of glycerol into the liver, indicating that 10H2DA promotes glycolysis and lipogenesis as an insulin‐like effect (Kameda et al., 1996). However, 10H2DA exerted different effects on PEPCK and G6Pase gene expression to those of insulin (Figure 4) even though previous findings showed that the insulin‐induced gene expression of AQP9 (Yokoyama et al., 2011) and these enzymes (Wolfrum et al., 2003) was regulated by Foxa2. Therefore, 10H2DA does not appear to exert the same effects as insulin and, thus, the mechanisms of action of 10H2DA in energy metabolism need to be elucidated in more detail in future studies.

Royal jelly, which is a viscous yellowish substance secreted from the hypopharyngeal and mandibular glands of worker honeybees, consists of 3%–6% lipids, which, in turn, consist of 80%–85% fatty acids, 4%–10% phenols, 5%–6% waxes, 3%–4% steroids, and 0.4%–0.8% phospholipids (Yonekura, 2008). Although 10H2DA possesses more than 30% fatty acids, it serves as a minor constituent in royal jelly (Lercker et al., 1981). The concentration of 10H2DA used in the present study to demonstrate the suppression of AQP9 gene expression was 2–4 mM, which was converted into 372–744 µg/ml. This concentration corresponds to 25–50 mg/ml royal jelly because the 10H2DA content of royal jelly is estimated to be approximately 0.7%–1.5%. Since the acid form of 10H2DA was used after neutralization with sodium hydroxide, its incorporation into cells may have been difficult. The 2‐decenoic acid ethyl ester was previously reported to be more efficient for the phosphorylation of ERK1/2, CREB, and Akt than the acid form in cultured neurons (Makino et al., 2010). On the other hand, GPR40–43, members of a subfamily of homologous G protein‐coupled receptors, were cloned downstream of CD22 on the human chromosomal locus 19q13.1 (Sawzdargo et al., 1997). Briscoe et al. (2003) identified medium and long chain fatty acids as ligands for the receptor GPR40/FFA1 (free fatty acid receptor 1) using ligand fishing experiments involving HEK293 cells expressing human GPR40. 10H2DA may function as a ligand for GPR40 and, if so, the affinity of 10H2DA to GPR40 may be extremely low.

The mechanisms by which 10H2DA promotes the phosphorylation of Akt and AMPK and Foxa2 gene expression currently remain unclear. We herein demonstrated that the suppression of AQP9 gene expression was partially recovered by the pre‐incubation of cells with Y‐27632 and palmostatin B, but not the Rac1 inhibitor or PI3K inhibitors (Figure 2c). Van der Heijden et al. (2008) proposed that the activation of RhoA/Rho kinase during ischemia–reperfusion (I/R) led to F‐actin rearrangements, which, in turn, facilitated reductions in PI3K and Akt activities and the induction of apoptosis. Simulated I/R also induced a decrease in phosphorylated Akt levels. The treatment with Y‐27632 maintained phosphorylated Akt levels and reduced the percentage of apoptotic nuclei during simulated I/R. The mechanism activating Akt with 10H2DA may be similar to that proposed by van der Heijden et al. Palmostatin B has been shown to inhibit APT1, specifically block Ras depalmitoylation, and then strongly induce the redistribution of the N‐Ras protein to endomembranes in MDCK cells (Dekker et al., 2010). Therefore, 10H2DA may respond to N‐Ras and phosphorylate Akt. Although the mechanisms by which 10H2DA activates Akt and AMPK have not yet been elucidated in detail, PI3K does not appear to be involved in this pathway.

10H2DA has been suggested to play a role in energy metabolism by suppressing AQP9 mRNA expression and this effect is similar to that of insulin; however, a high concentration of 10H2DA was needed to induce this effect in HepG2 cells. It is important to note that the effects of 10H2DA on PEPCK and G6Pase gene expression in glycometabolism differed from those of insulin. Hou et al. (2018) recently reported that AQP9 expression was significantly up‐regulated in type 1 and type 2 diabetes mellitus and suggested that insulin down‐regulated AQP9 by inhibiting phosphorylated JNK and activating phosphorylated p38. Further studies on the signal transduction pathway triggered by 10H2DA will contribute to our understanding of the effects of this acid in metabolism.

## CONFLICT OF INTEREST

The authors have no conflicts of interest associated with the study contents, which are solely of the responsibility of the authors and not necessarily representative of the official views of JSPS KAKENHI.

## ETHICAL APPROVAL

This study does not involve any human or animal testing.
